# Prevalence and determinants of anxiety and depression in long-term breast cancer survivors

**DOI:** 10.1186/s12888-022-03735-3

**Published:** 2022-02-09

**Authors:** Clara Breidenbach, Paula Heidkamp, Kati Hiltrop, Holger Pfaff, Anna Enders, Nicole Ernstmann, Christoph Kowalski

**Affiliations:** 1 German Cancer Society, Kuno-Fischer-Straße 8, 14057 Berlin, Germany; 2 Center for Health Communication and Health Services Research (CHSR), Department for Psychosomatic Medicine and Psychotherapy, University Hospital Bonn, Venusberg Campus 1, 53127 Bonn, Germany; 3 Institute of Medical Sociology, Health Services Research and Rehabilitation Science (IMVR), University of Cologne, Eupener Str. 129, 50933 Cologne, Germany; 4 Federal Centre for Health Education (BzgA), Maarweg 149-161, 50825 Cologne, Germany

**Keywords:** Anxiety, Depression, Breast cancer, Cancer survivorship, Hospital anxiety and depression scale

## Abstract

**Background:**

There is a significant number of long-term breast cancer survivors in Germany. However, research on the psychological challenges of cancer survivors is limited. This analysis describes prevalence, development and determinants of depression and anxiety 5 to 6 years after diagnosis and identifies predictors for an increase of anxiety and depression over time.

**Methods:**

Data from 164 women was collected by survey and tumour documentation during post-operative hospital stay, 40 weeks and 5 to 6 years after diagnosis. Anxiety and depression were measured by the Hospital Anxiety and Depression Scale. Sankey-diagrams were created for visual presentation of prevalence over time. Logistic and linear regression models were calculated to identify determinants of anxiety and depression.

**Results:**

Respondents had higher levels of depression and anxiety 5 to 6 years than 40 weeks after the diagnosis. Lower vocational status and having children were associated with depression, surgery type was correlated with anxiety, and age, as well as comorbidities, were predictors for both anxiety and depression 5 to 6 years after diagnosis. An increase of depression over time was more likely when having children and comorbidities. An increase in anxiety was less likely after cancer recurrence.

**Conclusions:**

Findings highlight that anxiety and depression are relevant burdens for breast cancer survivors in Germany. Several sociodemographic and clinical predictors are identified. There is need for psychosocial support after acute treatment and in the long-term. Research on psychological burdens of long-term breast cancer survivors in the identified vulnerable groups is needed.

## Background

In Germany, the 10-year breast cancer survival rate ranges from about 50% for men to about 70% for women [1]. As about 70,000 women and 700 men are diagnosed with breast cancer annually [1], there is a significant number of long-term breast cancer survivors in the German population. Hence, many of those affected need to cope with the long-term effects of breast cancer. There are a variety of difficulties breast cancer survivors have to face: employment and work-related issues [2], restrictions in quality of life as they often experience impaired physical, role, mental or cognitive functioning [3, 4], as well as fear of recurrence [5, 6]. Overall, the prevalence of psychological complaints is higher in former cancer patients than in non-affected reference populations [3, 7–11]. It has been shown that depressive symptoms and anxiety often remain beyond the treatment phase and are found in long-term survivors [7, 12–15].

The concept of cancer survivorship is gaining more attention, however, research on mental health problems and challenges in long-term survivors is still limited compared to the phases of diagnosis and acute treatment [3, 16, 17]. Several reviews state a need for research regarding cancer survivorship and psychological challenges, especially the later years (from 5 years after a cancer diagnosis) including the identification of risk factors [3, 8, 17]. It has been indicated that depression and anxiety may be a factor in predicting breast cancer recurrence and survival [18]. Depression and anxiety in breast cancer patients in an acute treatment phase have been related to several factors [16]. For example, low emotional and social support have been associated with higher risk for depression or anxiety [19]. Physical symptoms and impairments have been related to depression and anxiety in metastazised breast cancer patients [20]. For breast cancer patients undergoing chemotherapy demographic factors such as education, age and gender as well as economic factors such as unemployment as well as psycosocial factors such as self-efficacy or perceived stress may be linked to depression [21].

This analysis aims to examine the prevalence and development of depression and anxiety 5 to 6 years after diagnosis compared to 40 weeks after diagnosis. Further, it aims to investigate a) predictors for an increase of depressive and anxiety symptoms over time and b) determinants of depression and anxiety in long-term breast cancer survivors 5 to 6 years after the diagnosis. Findings may contribute to a more tailored psycho-oncological care for long-term survivors by identifying risk groups at an early stage and designing preventive measures.

## Methods

### Data collection

The present analysis was carried out as part of the research project B-CARE (Breast Cancer Patients’ Return to Work) funded by the Deutsche Rentenversicherung Bund (German Federal Pension Insurance). B-CARE is a mixed-methods study that was initiated to examine socio-demographic and psychosocial determinants of the use of rehabilitation services as well as determinants of occupational reintegration after breast cancer [22]**.** In order to recruit study participants, 530 patients who had participated in the PIAT study (Strengthening patient competence: Breast cancer patients’ information and training needs, funded by the German Federal Ministry of Health) in 2013 and who were employed at the time of their breast cancer diagnosis in 2013/14, were asked per mail to complete a follow-up questionnaire 5 to 6 years after diagnosis in 2019. Inclusion criteria for the PIAT study were an initial breast cancer diagnosis and surgery in a German Cancer Society-certified breast cancer centre between 1 February and 31 August, 2013. For further information on the PIAT study see for example Halbach et al. [23, 24]. Participant recruiting and data collection for B-CARE was conducted by the study director and team (NE, PH, KH). A subsample of the PIAT sample that filled in written consent and the B-CARE questionnaire was included in the B-CARE study. A subset of participants that filled in the questionnaire for B-CARE was also invited for semi-structured interviews. Data from the B-CARE survey 5 to 6 years after diagnosis (T4) were then linked with data from the PIAT study from 2013 to allow for a consideration of four measurement points over 5 years (T1: during post-operative hospital stay; T2: 10 weeks after diagnosis; T3: 40 weeks after diagnosis). In the PIAT study, survey data were linked with clinical and treatment data documented by the hospital (see section “Variables”). B-CARE has been approved by the ethical committee of the University Hospital Bonn (316/18).

### Variables

The following data were used for the current analyses:

There are two dependent variables in this study, anxiety as well as depression, which were operationalised according to the Hospital Anxiety and Depression Scale (HADS), measured 5 to 6 years (T4) and 40 weeks (T3) after diagnosis. The HADS measures anxiety and depression, with seven items for each construct. According to Herrmann et al. [25], the seven items were summarised as scores for each construct. In this study, scores between zero and under eight are assessed as “no anxiety” or “no depression”, scores between eight and under 11 as a “mild anxiety” or “mild depression”, scores between 11 and under 15 as a “moderate anxiety” or “moderate depression” and scores from 15 as “severe anxiety” or “severe depression” [25]. The HADS has been applied widely and tested for validity and accuracy [26, 27] and is recommended in the German clinical psycho-oncology guideline as one of two instruments for the assessment of psychosocial burden ([28], p. 49). It is thus widely used by psycho-oncologists not only for research but also in routine practice.

Independent variables from survey data were age as a categorical variable (under 50 years, 60 to 69 years, 70 to 79 years), vocational training (no vocational qualification, general vocational training, specialised training or training for master craftsmanship, university (of applied sciences) degree), living together with a partner (yes/no), children (yes/no), number of comorbidities (0, 1, 2 and more) and cancer recurrence (yes/no). Independent variables from tumour documentation systems in the breast cancer centres were UICC TNM stage (0, 1, 2, 3 and 4) and type of surgery (breast-conserving surgery, mastectomy).

### Data analysis

First of all, descriptive statistics were calculated in order to describe the sample characteristics. Secondly, paired sample t-tests were performed that tested to what extent mean scores of depression and anxiety vary between T3 and T4. Effect size Cohen’s d was calculated for the t-tests. Above, Sankey diagrams were created using the R program (“networkD3” package), in order to display how respondents’ depression/anxiety levels changed over time. Subsequently, regression analyses were performed in order to identify predictors for anxiety/depression. Firstly, binominal logistic regression models were calculated in order to identify respondents’ characteristics associated with shifting to a higher level of depression or anxiety according to the classification provided by Hermann et al. [25] from T3 measurement to T4 measurement. Therefore, dummy variables were created as dependent variables, coding 0 for respondents that improved or stayed at the same levels of anxiety or depression, respectively, and coding 1 for respondents that shifted to a higher level. Secondly, linear regression models were calculated in order to identify determinants for depression and anxiety at measurement T4. Only cases with valid anxiety/depression scores at T3 and T4 were included in the analyses. Missing values for the independent variables were included as separate categories in order to prevent case exclusion as well as to control for potential effects. Missing categories were excluded from the logistic regression analysis when cases in one category did not vary in the dependent variable. Independent variables for the models were chosen by theoretical considerations. Then, univariate linear (dependant variables: anxiety or depression 5 to 6 years after diagnosis) and logistic (dependant variables: increase in anxiety or depression) regressions were calculated for each independent variable. Afterwards, the variables were added stepwise to the models while monitoring the variables’ coefficients/odds ratios, *p*-values and confidence intervals as well as the models R^2^/ Nagelkerke’s-R^2^ and McFadden’s R^2^ and Aikaike Information Criterion in order to check confounding effects and model accuracy. For all statistical analyses, except for the Sankey diagrams, STATA/IC 15.1 was used.

## Results

### Sample

Table 1 summarises the describtive sample characteristics. The 164 women that submitted a questionnaire were 57 years old on average at T4 (Standard Deviation (SD): 6.8; min.-max.: 36–79). The majority of the respondents (56.1%) stated that their highest level of vocational qualification was general vocational training. Most respondents lived with a partner (80.5%) and stated that they had children (79.9%). The majority was assigned to the first (39.6%) or second UICC stage (32.9%) during their post-operative hospital stay (T1). Most participants (73.2%) received breast-conserving surgery. Of the 164 respondents, 74 (45.1%) stated that they had no other disease besides cancer, 50 persons (30.5%) named one comorbidity and 40 persons (24.4%) named two or more diseases besides cancer. Thirty-four respondents (20.7%) indicated cancer recurrence after their initial breast cancer diagnosis.

**Table 1:** Descriptive sample characteristics

Variable	Time of measurement^**a**^, source	Options	n (%)
Anxiety	T4, survey	n (%)	164 (100)
		Mean (standard deviation)	8.4 (2.0)
		Range	3.5–14
	T3, survey	n (%)	164 (89.1)
		Mean (standard deviation)	6.0 (3.8)
		Range	0–18
Depression	T4, survey	n (%)	164 (100)
		Mean (standard deviation)	7.5 (1.9)
		Range	4–15
	T3, survey	n (%)	164 (89.1)
		Mean (standard deviation)	3.1 (3.1)
		Range	0–15
Age	T4, survey	Under 50 years	17 (10.4)
		50 to 59 years	91 (55.5)
		60 to 69 years	49 (29.9)
		70 to 79 years	5 (3.1)
		Missing	2 (1.2)
Vocational training	T1, survey	No vocational training	6 (3.7)
		General vocational training	92 (56.1)
		Specialised training or training for master craftsmanship	18 (11.0)
		University (of applied sciences) degree	46 (28.1)
		Missing	2 (1.2)
Living together with a partner	T4, survey	No	31 (18.9)
		Yes	132 (80.5)
		Missing	1 (0.6)
Children	T4, survey	No	27 (16.5)
		Yes	131 (79.9)
		Missing	6 (3.7)
UICC TNM stage	T1, clinical tumor documentation	0	11 (6.7)
		1	65 (39.6)
		2	54 (32.9)
		3 and 4	11 (6.7)
		Missing	23 (14.0)
Type of surgery	T1, clinical tumor documentation	Breast-conserving surgery	120 (73.2)
		Mastectomy	32 (19.5)
		MIssing	12 (7.3)
Number of comorbidities	T4, survey	0	74 (45.1)
		1	50 (30.5)
		2 and more	40 (24.4)
Cancer recurrence	T4, survey	No	129 (78.7)
		Yes	34 (20.7)
		Missing	1 (0.6)

^a^Time of measurement: T1 = during post-operative hospital stay; T2 = ten weeks after diagnosis; T3 = 40 weeks after diagnosis; T4 = five to six years after diagnosis

### Prevalence of depression and anxiety

**Fig. 1:**
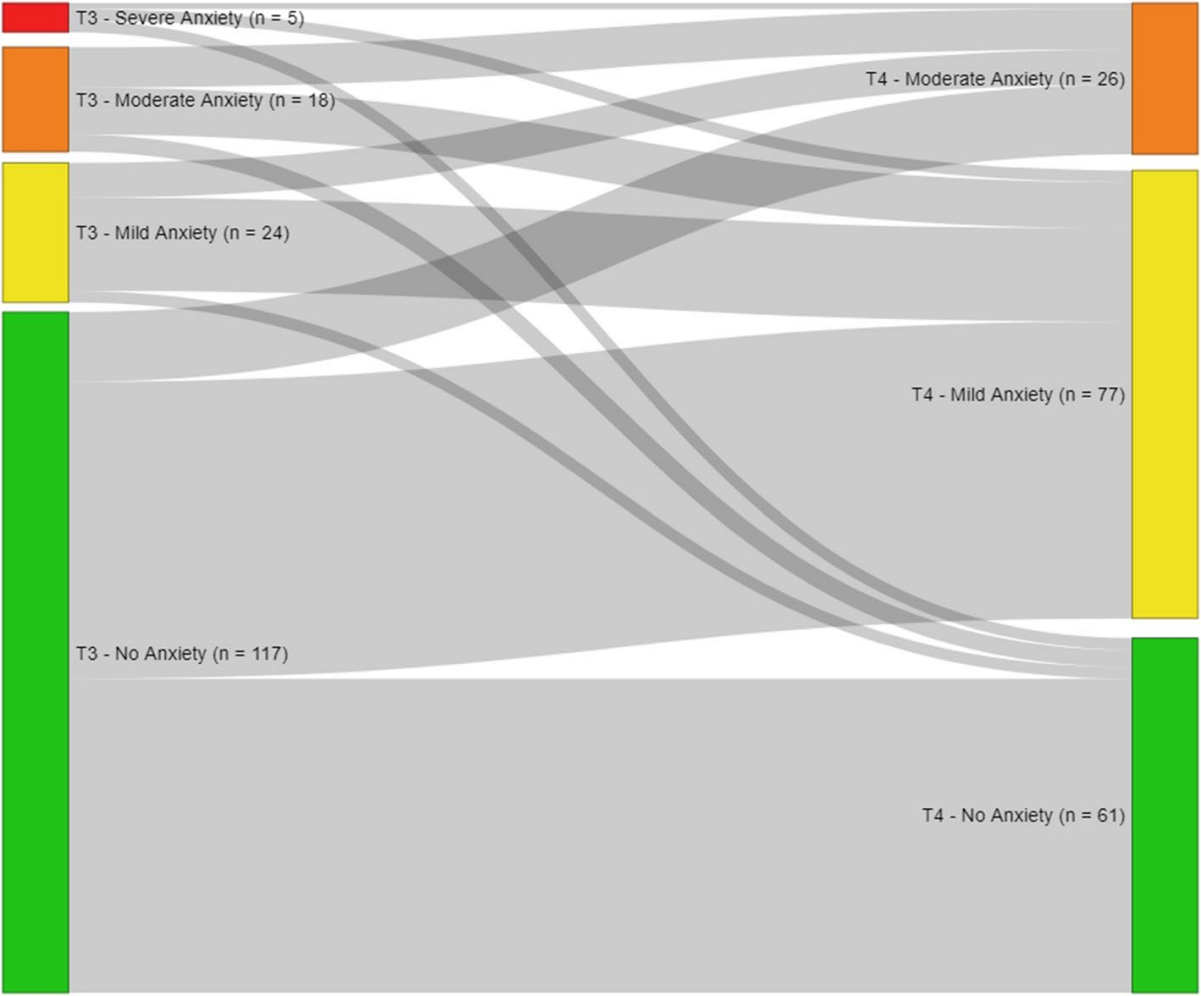
Sankey-diagram showing the respondents’ transfers between anxiety levels at T3 and T4 (*n* = 164); the thicker the grey line, the more patients

Five to six years after the diagnosis (T4), the respondents had an average anxiety score of 8.4 (SD: 2.0), which is on the mild anxiety level. Forty weeks after the diagnosis (T3), the average score was significantly lower at 6.0 (SD: 3.8) (t = 8.4961, *p* < .001, d = 0.66, *n* = 164) indicating no anxiety. About 34% (*n* = 56) of the respondents shifted to a higher level of anxiety over time (Fig. 1, Table 1).

**Fig. 2:**
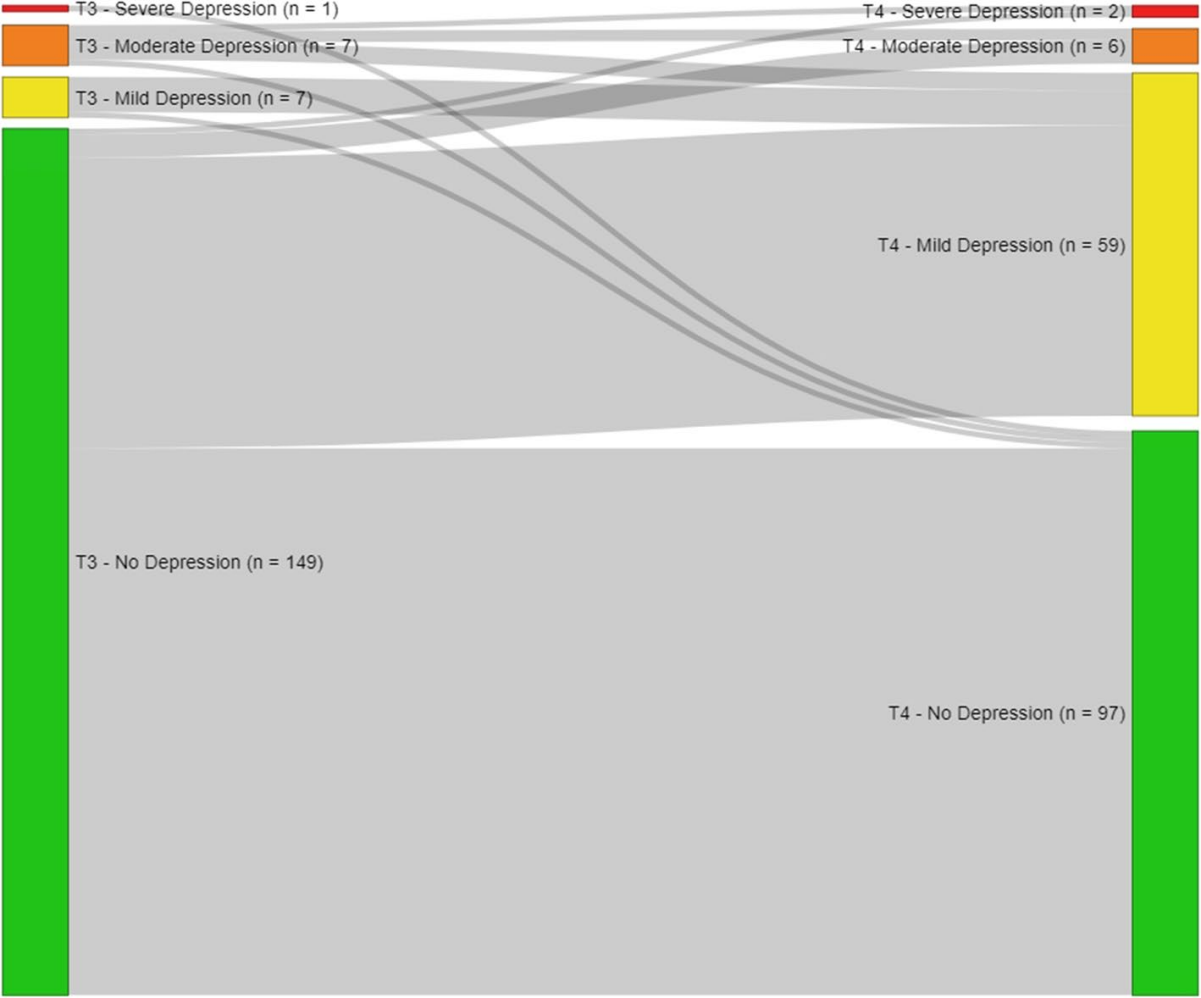
Sankey-diagram showing the respondents’ transfers between depression levels at T3 and T4 (*n* = 164); the thicker the grey line, the more patients

For depression, respondents had a significantly higher average score of 7.5 (SD: 1.9) 5 to 6 years after diagnosis (T4) than 40 weeks after diagnosis (Mean: 3.1, SD: 3.1; t = 19.1236, *p* < .001, d = 1.49, *n* = 164). At T3 measurement, 149 (90.9%) respondents had no depressive symptoms. Of these, 34.9% showed mild to severe depressive symptoms at measurement time T4. Forty-two per cent of the respondents (*n* = 69) shifted to a higher level of anxiety over time (Fig. 2, Table 1).

### Multivariable analyses

Binominal logistic regression models were used to identify respondents’ characteristics associated with shifting to a higher level of depression or anxiety in measurement T4 compared to T3 measurement. Model 1.1 (see Table 2) shows that respondents with children (Odds Ratio (OR) = 9.5 (1.87, 48.40), *p* = .007) and with two or more comorbidities (OR = 3.75, (1.38, 10.18), *p* = .01) were more likely to have increased levels of depression over time. When not controlling for having children and comorbidities, UICC TNM stage 0 (OR = 4.27, (1.09, 17.15), *p* = .04) as well as 3 and 4 (OR = 4.66, (1.06, 19.09), *p* = .04) are significantly associated with an increase of depression over time. The overall logistic regression model 1.1 was statistically significant, χ2(17) = 32.78, *p* = .01, with Nagelkerke’s Pseudo-R^2^ = 0.27, McFadden’s R^2^ = 0.17 and *n* = 153.

**Table 2:** Results of logistic regression analyses for the increase of depression (Model 1.1) and an increase of anxiety (Model 1.2)

Variable	Options	Model 1.1. Increase Depression			Model 1.2. Increase Anxiety		
		Odds ratio	***p***-value	95% CI	Odds ratio	***p***-value	95% CI
Intercept		0.05	.002	0.01	0.53	.32	0.15 1.89
Age	Under 50 years	1.40	.62	0.37 5.28	0.30	.08	0.08 1.13
	50 to 59 years	Reference					
	60 to 69 years	0.59	.27	0.23 1.50	0.55	.17	0.24 1.28
	70 to 79 years	0.38	.50	0.02 6.25	0.27	.31	0.02 3.36
	Missing				1.20	.91	0.06 24.12
Vocational training	No vocational training	2.76	.30	0.40 19.16	0.70	.71	0.11 4.57
	General vocational training	Reference					
	Specialised training or training for master craftsmanship	0.83	.77	0.23 2.94	0.63	.44	0.20 2.04
	University (of applied sciences) degree	0.48	.15	0.18 1.31	0.68	.37	0.29 1.58
	Missing						
Living together with a partner	No	Reference					
	Yes	1.03	.96	0.36 2.96	1.17	.74	0.45 3.02
	Missing						
Children	No	Reference					
	Yes	9.53	.007	1.87 48.40	2.38	.11	0.86 6.81
	Missing	Reference			1.93	.56	0.21 17.92
UICC TNM stage	0	2.33	.27	0.51 10.57	1.05	.95	0.25 4.29
	1	Reference					
	2	0.88	.79	0.36 2.19	1.00	1.00	0.44 2.29
	3 und 4	2.45	.25	0.56 11.18	0.58	.47	0.13 2.53
	Missing	0.83	.84	0.15 4.72	0.45	.29	0.10 2.01
Type of surgery	Breast-conserving surgery	Reference					
	Mastectomy	1.25	.66	0.46 3.44	1.71	.25	0.69 4.25
	Missing	0.61	.64	0.08 3.77	5.70	.06	0.91 35.87
Number of comorbidities	0	Reference					
	1	1.05	.92	0.41 2.71	0.90	.80	0.39 2.06
	2 and more	3.75	.01	1.38 10.18	1.15	.76	0.47 2.81
Cancer recurrence	No	Reference					
	Yes	1.57	.33	0.64 3.86	0.39	.04	0.16 0.97
	Missing						
n		153			160		
Nagelkerke’s-R^2^		0.27			0.15		
McFadden’s R^2^		0.17			0.08		

Predictors of higher depression/anxiety scores, 5 to 6 years after diagnosis

Regarding anxiety, cancer recurrence is associated with a decrease in anxiety over time (OR = 0.39 (0.16, 0.97), *p* = .04). However, this association is only significant when controlling for having children and type of surgery. Model 2.1 could not reach significance in total, χ2(19) = 18.23, *p* = .51.

Linear regression models were used to identify determinants for depression and anxiety at measurement T4. Model 2.1 (Table 3) with depression as dependent variable indicates that respondents under 50 years showed significantly higher values for depression than respondents in the reference group of 50 to 59 years (Coef. = 1.17 (0.21, 2.12), *p* = .02). Moreover, lower depression values were found in respondents with a university (of applied sciences) degree than respondents with a general vocational training (Coef. = − 1.15 (− 1.83, − 0.47), *p* = .001). Having children was associated with higher depression (Coef. = 1.17 (0.35 1.98), *p* = .01). Respondents with two or more comorbidities showed higher levels of depression than survivors without comorbidities (Coef. = 1.31 (0.58, 2.03), *p* < .001). Model 2.1 explains about 32% of the variance in depression (*n* = 164) and is statistically significant (F(22, 141) = 3.05, *p* < .001).

**Table 3:** Results of linear regression analyses for depression (Model 2.1) and anxiety (Model 2.2) 5 to 6 years after diagnosis

Variable	Options	Model 2.1. Depression			Model 2.2. Anxiety		
		Coefficient	***p***-value	95% CI	Coefficient	***p***-value	95% CI
Intercept		6.43	.001	5.45 7.42	7.57	.001	6.49 8.65
Age	Under 50 years	1.17	.02	0.21 2.12	1.08	.04	0.03 2.12
	50 to 59 years	Reference					
	60 to 69 years	0.41	.23	−0.27 1.09	− 0.74	.05	−1.48 0.00
	70 to 79 years	−0.33	.72	−2.18 1.51	−1.89	.07	−3.90 0.13
	Missing	−0.97	.44	−3.47 1.53	−1.04	.45	−3.76 1.69
Vocational training	No vocational training	1.01	.18	−0.48 2.50	0.31	.70	−1.31 1.94
	General vocational training	Reference					
	Specialised training or training for master craftsmanship	0.02	.97	−0.90 0.93	0.81	.11	− 0.20 1.81
	University (of applied sciences) degree	−1.15	.001	−1.83 -0.47	−0.27	.47	−1.01 0.47
	Missing	0.47	.72	−2.13 3.06	1.21	.40	−1.62 4.04
Living together with a partner	No	Reference					
	Yes	−0.67	.08	−1.43 0.91	−0.34	.42	−1.17 0.49
	Missing	1.95	.36	−2.20 6.09	−1.46	.52	−6.00 3.06
Children	No	Reference					
	Yes	1.17	.01	0.35 1.98	0.84	.06	−0.04 1.73
	Missing	1.53	.09	−0.25 3.30	0.63	.52	−1.31 2.56
UICC TNM stage	0	0.06	.92	−1.11 1.24	−0.23	.72	−1.52 1.05
	1	Reference					
	2	0.06	.86	−0.62 0.74	0.14	.71	−0.60 0.88
	3 und 4	0.20	.74	−1.00 1.39	−0.59	.38	−1.89 0.72
	Missing	0.57	.30	−0.52 1.67	0.12	.84	−1.08 1.32
Type of surgery	Breast-conserving surgery	Reference					
	Mastectomy	0.52	.16	−0.21 1.26	0.91	.03	0.10 1.71
	Missing	−1.21	.09	−2.62 0.20	0.46	.55	−1.08 2.00
Number of comorbidities	0	Reference					
	1	0.57	.09	−0.09 1.24	0.43	.25	−0.30 1.15
	2 and more	1.31	.001	0.58 2.03	0.94	.02	0.15 1.73
Cancer recurrence	No	Reference					
	Yes	0.13	.71	−0.56 0.82	−0.29	.44	−1.05 0.46
	Missing	−1.35	.45	−4.88 0.82	−0.23	.91	−4.08 3.62
n		164			164		
R^2^		0.32			0.20		

The linear regression model regarding anxiety, model 2.2, showed that respondents under 50 years old show higher levels of anxiety than the reference group (Coef. = 1.08 (0.03, 2.12), *p* = .04) as well as those with comorbidities (two or more vs. no comorbidities; Coef. = 0.94 (0.15, 1.73), *p* = .02). However, the significant correlation with comorbidities only applies when controlling for age. Receiving a mastectomy was associated with higher anxiety scores than receiving breast-conserving surgery (Coef. = 0.91 (0.14, 1.71), *p* = .03) Model 2.2 is significant (F(22, 141) = 1.65, *p* = .04) and explains about 20% of the variance of anxiety at T4 (*n* = 164).

## Discussion

The objective of this analysis was to describe the prevalence, development and determinants of depression and anxiety in long-term breast cancer survivors in Germany. The findings reveal that survivors show significantly higher depression and anxiety scores 5 to 6 years after diagnosis than 40 weeks after diagnosis. According to Cohen [29], the effect sizes of these findings are medium to large. As the Sankey-diagrams demonstrate, about one-third of the respondents recorded a change to a higher level of depression over time, and more than one third shifted to a higher level of anxiety. In total, the sample showed higher values for depression and anxiety 5 to 6 years after diagnosis than women in the German general population ([30]: anxiety 5.0, depression 4.7). An explanation for these detected trajectories might be that 40 weeks after the diagnosis, positive emotions, like relief and appreciation of life prevail, because of the illness that had just been conquered [31]. In comparison, 5 to 6 years after the diagnosis, women have to deal with the emotional, social, financial and physical long-term effects of their breast cancer diagnosis, which might lead to more anxiety and depression. The literature on the topic is sparse, however, it is acknowledged that depression and anxiety are serious issues for breast cancer survivors and should be addressed [7, 12, 32, 33].

Multivariable analyses revealed patient characteristics that were significantly associated with higher levels of psychological burden 5 to 6 years after diagnosis. Age was reported to be associated with depression and anxiety: Respondents younger than 50 years were more distressed than survivors in their fifties. Research has shown that the variation of the psychologic impact of cancer is related to age, in that older persons are often less affected [7, 32, 33]. Receiving a cancer diagnosis at a younger age often relates to a better prognosis, however, it might also question feelings of security and controllability, e.g., regarding reproductive concerns [34]. Consistent with the literature [7], we found vocational training level to be a significant predictor for depression 5 to 6 years after diagnosis.

Furthermore, having two or more comorbidities was found to be associated with the level of depression and anxiety 5 to 6 years after the diagnosis, as well as an increase of depression over time. This finding is consistent with previous findings [12, 17] and might be explained by the fact that better physical health may help to manage daily requirements as well as to rebuild structure and normalcy to daily life after the active treatment phase.

Our analysis indicates that having children correlates with an increase of depression over time, which has been reported before [35, 36]. A review by Semple and McCane [37] highlights that parents with cancer might struggle to talk to their children about cancer, experience feelings of failure as a parent or perceive an increased effort in order to maintain routines at home for their children.

Mastectomy in our analysis is related to higher anxiety scores 5 to 6 years after the diagnosis compared to survivors with breast-conserving surgery which is in line with previous research [38] and might be related to body image issues [39] and pain [40]. Cancer recurrence, in turn, is associated with a decrease in anxiety over time. This finding might be explained by illness trajectories in chronic illnesses [41, 42]: If cancer recurs or progresses, coping phases might start over such as shock, defence mechanisms, anger and acceptance. However, the HADS only measures anxiety that someone would admit to oneself. Thus, if women are in a phase of defence due to a recurrence of their disease, they might not admit anxiety to themselves, and it might not be detected. Moreover, the model investigating determinants for an increase of anxiety could not reach significance, which indicates that the major predictor for an increase of anxiety of time is not included in this analysis and results should be interpreted carefully.

Regarding the limitations of the study, these analyses are based on an observational study design that does not allow any causal interpretations. Moreover, the sample of this analysis has a slightly higher proportion of females with a university (of applied science) degree and a lower proportion of females without vocational training than in the general German population [43]. Moreover, there might be a bias in the sample regarding healthier and more motivated cancer survivors or regarding less survivors with a migration background/lack of German language skills. Future studies should recruit more groups of long-term survivors with a lower level of education or migration background in order to obtain a more distinct picture of mental health problems in these risk groups, e.g., with qualitative approaches for survivors that may experience troubles with paper-based surveys [3, 8, 17]. On top of that, data for this analysis did not provide information about change in socioeconomic status over time, which could be of interest for future research. Furthermore, no men were included in the current analysis. Only about 1 % of all breast cancer diagnoses are made in men [1]. Due to a lack of care structures, there are many uncertainties in male breast cancer patients [44], which is why future research projects should include long-term male survivors of breast cancer. Moreover, depression and anxiety were measured with only one instrument (HADS) in this study. The HADS has been widely applied and validated in many languages, however, it has also been subject of discussion, especially in terms of its current thresholds [45]. While testing the prerequisites for the linear regression model, scatter plots revealed a light violation of the assumption of homoscedasticity, suggesting that the model is suited better for predicting lower depression levels.

## Conclusion

Overall, the results suggest that anxiety and depression are a serious psychological burden for long-term breast cancer survivors in Germany. Findings emphasise the need for psychological and social support services after acute treatment and in the long-term. Particular attention should be given to younger survivors, to those with children, to those with comorbidities, those with a lower level of professional training as well as to those undergoing mastectomy. Further research on the psychological burdens of long-term breast cancer survivors in the identified vulnerable groups is urgently needed in order to tailor support services and target risk groups. Moreover, future research should investigate whether survivors in need utilise counselling services and identify inhibiting and facilitating factors for the utilisation.

## Data Availability

According to the patient consent form data is not available for scientific use by others than the project group members.

## Abbrevations

B-CARE: Breast Cancer Patients’ Return to Work
HADS: Hospital Anxiety and Depression Scale
PIAT: Strengthening patient competence: Breast cancer patients’ information and training needs
SD: Standard Deviation
OR: Odds Ratio
